# Measuring instrument: knowledge, attitudes and practices of people with pulmonary tuberculosis

**DOI:** 10.1590/1518-8345.2608.3086

**Published:** 2019-01-07

**Authors:** Alba Idaly Muñoz-Sánchez, Yurian Lida Rubiano-Mesa, Carlos Julio Saavedra-Cantor

**Affiliations:** 1 Universidad Nacional de Colombia, Facultad de Enfermería, Bogotá, DC, Colombia.

**Keywords:** Tuberculosis, Patients, Health Knowledge, Attitudes, Practice, Validation Studies, Psychometrics, Nursing Assessment, Tuberculose, Pacientes, Conhecimentos, Atitudes e Prática em Saúde, Estudos de Validação, Psicometria, Avaliação em Enfermagem, Tuberculosis, Pacientes, Conocimientos, Actitudes y Práctica en Salud, Estudios de Validación, Psicometría, Evaluación en Enfermería

## Abstract

**Objective:**

to build an instrument to attain reliable and valid measurements of the knowledge, attitudes and practices of patients with pulmonary tuberculosis.

**Methods:**

methodological study that measured the sensitivity, reliability and validity of the instrument content. Studies of reliability and content validity comprehensibility involved 234 patients with pulmonary tuberculosis.

**Results:**

an integrative review was conducted for theoretical foundation. The sensitivity study comprised 30 patients with pulmonary tuberculosis, who had greater knowledge on tuberculosis (12.03) than the control group (9.93). Factor analysis showed that 7 factors explained 67.8% of the variance. Content validity identified a 98.3 % comprehensibility, and the expert trial assessed the sufficiency, clarity, relevance and coherence criteria, showing agreement between judges.

**Conclusions:**

the instrument has studies of sensitivity, reliability and content validity that showed it can be applied to patients with pulmonary tuberculosis; nevertheless, cultural and semantic adaptations must be developed for other scenarios.

## RESUMO

**Objetivo:**

elaborar um instrumento que permita obter medidas confiáveis e válidas sobre os conhecimentos, atitudes e práticas de pacientes com tuberculose pulmonar.

**Métodos:**

estudo metodológico que avalia a sensibilidade, a confiabilidade e a validade do conteúdo do instrumento. Para avaliar a confiabilidade e a compreensibilidade da validade de conteúdo, participaram deste estudo 234 pacientes com tuberculose pulmonar.

**Resultados:**

realizou-se uma revisão integrativa como fundamentação teórica. No estudo da sensibilidade, participaram 30 pacientes com tuberculose pulmonar que tinham mais conhecimentos sobre a tuberculose (12,03) do que o grupo controle (9,93). Na análise fatorial verificou-se 7 fatores que explicavam 67,8% da variância. Na validade de conteúdo identificou-se uma compreensibilidade de 98,3%, e, na avaliação dos especialistas, analisou-se os critérios de suficiência, clareza, pertinência e coerência, que mostraram concordância entre os especialistas.

**Conclusões:**

o instrumento conta com estudos de sensibilidade, confiabilidade e validade de conteúdo que mostraram ser possíveis de aplicar em pacientes com tuberculose pulmonar; no entanto, é necessário desenvolver adaptações culturais e semânticas em outros contextos.

## RESUMEN

**Objetivo:**

construir un instrumento que permita obtener medidas confiables y válidas sobre los conocimientos, actitudes y prácticas de los pacientes con tuberculosis pulmonar.

**Métodos:**

estudio metodológico que midió la sensibilidad, confiabilidad y validez de contenido del instrumento. En los estudios de confiabilidad y comprensibilidad de validez de contenido participaron 234 pacientes con tuberculosis pulmonar.

**Resultados:**

se realizó una revisión integrativa para la fundamentación teórica. En el estudio de sensibilidad participaron 30 pacientes con tuberculosis pulmonar que tenían mejores conocimientos sobre la tuberculosis (12,03) que el grupo control (9,93). En el análisis factorial se evidenciaron 7 factores que explicaban el 67,8 % de la varianza. En la validez de contenido se identificó una comprensibilidad del 98,3 % y en el juicio de expertos se evaluaron los criterios de suficiencia, claridad, relevancia y coherencia, los cuales mostraron concordancia entre los jueces.

**Conclusiones:**

el instrumento cuenta con estudios de sensibilidad, confiabilidad y validez de contenido que evidenciaron que puede ser aplicado a los pacientes con tuberculosis pulmonar; no obstante, es necesario desarrollar adaptaciones culturales y semánticas para otros escenarios.

## Introduction 

Tuberculosis (TB) is an infectious disease of global range, caused by the *Mycobacterium tuberculosis*, or Koch bacillus. The World Health Organization (WHO) stresses, through the *Global Tuberculosis Report 2017*, that 10.4 million people around the world contracted TB in 2016^1^. In Colombia, during 2016, 13,626 TB cases were assessed^2^, being 1,327 in the city of Bogotá^3^. 

Knowledge, Attitudes and Practices (KAP) on TB comprise aspects such as what is the disease, the transmission mechanism, risk factors, signs and symptoms, diagnosis, treatment, and prevention. Knowledge is the ideas, information, and beliefs a person has from factors such as socialization, experiences, culture, and information access, being thus a fundamental component of the CAP since it may affect attitudes, as it defines preferences and predisposes to actions. Attitudes are predispositions, values, and emotions people have when facing a situation; and practices are actions performed, which stem from the family context, knowledge, attitudes, habits and customs, among others, that one may have before a health phenomenon such as the TB^4^ ^-^ ^6^. 

The Collective Health and Care Research Group of the Nursing School of the National University of Colombia has previously conducted an integrative review aimed at describing the scientific production of tools and instruments for assessing knowledge on TB and that are available for the health personnel, patients, community and family; it was found that there are no reliable and valid instruments to be applied in patients with Pulmonary Tuberculosis (PTB)^7^. In addition, the development of an instrument on the KAP of patients with PTB allows one to establish a systematic and rigorous process, in a way that results are measurable, reliable, and valid^8^. It should be mentioned that the WHO, through the *”End of TB” Strategy Post 2015,* proposes in Pillar III - which is related to scientific research and innovation - the importance of research in optimizing the execution and impact of the resources of TB control programs, so that the construction of an instrument on the KAP of patients with PTB seeks to subsequently generate the measurement of educational interventions that strengthen the adherence to anti-tuberculosis treatment and, thus, contribute to the control of tuberculosis through health education^9^. 

In this sense, the adequate knowledge of TB patients about their disease can promote adherence to tuberculosis treatment and, therefore, avoid the appearance of drug-resistant forms of TB; promote the identification of contacts of people with TB; strengthen the early detection of the disease and the demand of health services for new cases; promote assistance to controls with the health team and the performance of bacteriological controls; avoid practices of self-stigmatization, such as isolation in rooms of the dwelling and not sharing food and utensils with the family, and may even transform the inadequate attitudes and practices of TB patients about their disease^10^ ^-^ ^11^. 

To analyze the KAP, one needs to develop reliable and valid instruments that allow the systematic observation of health phenomena from the definitions of the construct, its dimensions and items^12^. This study aims at building an instrument that allows one to obtain reliable and valid measures of the KAP of PTB patients on their illness. 

The Standards for Educational Psychological Testing are considered in building such instrument. The development of measuring instruments includes the theoretical foundation, systematic observation of the phenomenon of interest, and empirical studies to measure the sensitivity, reliability and validity of such tool^8^. 

## Methods

**Figure 1:**
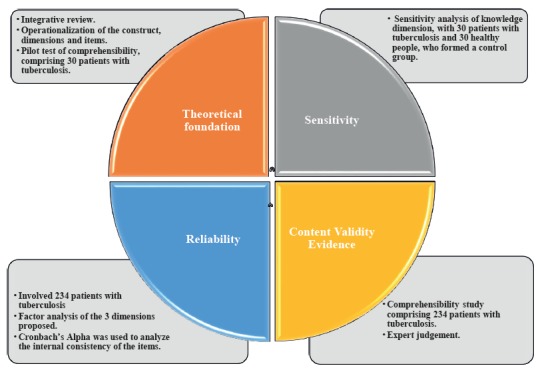
Phases of the building process of the Instrument Knowledge, Attitudes and Practices of patients with pulmonary tuberculosis, Bogotá, Colombia, 2017

Methodological study that measured the sensitivity, reliability and validity of the content within a measuring instrument^8^. Figure 1 describes the phases developed by the study.

In phase 1, the theoretical foundation was developed to define the variables associated with the KAP of PTB patients on their illness, to make a structure measuring key aspects of the phenomenon and to direct the development of items^8^. The KAP construct was operationalized in PTB patients on their disease, based on an integrative review including: delineation of the objective and problem of the review; definition of criteria for the inclusion and exclusion of articles; search in the databases Virtual Health Library (VHL), Embase, PubMed, Scielo, Science Direct and Web of Science; critical evaluation of the studies; categorization and analysis of the selected ones^13^. 

The integrative review aimed at describing the theoretical and conceptual development in building an instrument with reliable and valid measures of the KAP of PTB patients. Articles published in indexed journals from January 1, 2010, to August 31, 2016, and which included the KAP of PTB patients on their health/illness process were included. Articles that dealt with the KAP of patients with extrapulmonary TB were excluded. 

The Descriptors in Health Sciences (DeCS) used were: Knowledge Attitudes and Practices in Health; Tuberculosis; Patients. The Medical Subject Headings (MeSH) used were: Health Knowledge, Attitudes, Practice: Tuberculosis; Patients. Different search equations were performed with the previously described descriptors. Subsequently, articles were critically evaluated by the researchers and the classification of evidence levels proposed by the Scottisch Intercollegiate Guidelines Network (SIGN) were used^14^. Once the articles were classified based on the level of scientific evidence according to the SIGN, a second reading was carried out to categorize the dimensions and subdimensions of the measuring instrument in order to identify the KAP of TB patients, purpose for which a document was constructed with the variables: database; year; continent and/or region; country; authors; article title; objective; type of study; whether the study included only the KAP of TB patients, given that several studies were found that addressed KAP of TB patients and/or their relatives and community; information means through which they acquired their KAP on TB; knowledge about TB; attitudes about TB; TB practices; level of evidence of the article according to the SIGN, and some general observations.

Consecutively, the dimensions and items of the instrument were defined, and a version was devised to be applied in a pilot test with 30 PTB patients from a Bogotá health institution, to analyze its comprehensibility. Patient inclusion criteria were: people with PTB who received treatment by the TB Program of Bogotá, people newly admitted into the program, and those aged more than 18 years old, who agreed to participate trough a written informed consent form. The exclusion criteria were: people with cognitive disabilities and people in readmission modality due to abandonment or relapse.

In phase 2, a sensitivity analysis was conducted on the knowledge dimension of the instrument, with 30 PTB patients and 30 people forming a control group^15^. The inclusion and exclusion criteria of the patients’ group were the same already mentioned for the pilot test of comprehensibility. The inclusion criteria of the control group were: people who were in waiting rooms of the health institutions at the time of instrument application; who had not had TB or a family member with the disease since this may affect their knowledge on the illness; people aged more than 18 years old who agreed to participate through a written informed consent form. People with cognitive disabilities were excluded. 

Responses of the items were functionally analyzed from the psychometric model proposed by Sperman, which assumes that the number of correct responses is proportional to the attribute evaluated. Difficulty and discrimination parameters were calculated and compared. Difficulty indicated the level of knowledge of the groups regarding the information asked. This item was calculated from the percentage of participants who responded it correctly; an analysis that allowed comparing the number of correct answers in each group, which showed values ranging from 0 to 1 - values close to 1 indicated the item was more difficult for the group of patients with PTB or for the control group. Discrimination is a statistical parameter that is related to the ability of the items to be answered by people who dominate the information and by those who guess randomly without knowing the subject, it was calculated considering low and high performance groups: the values ranged between -1 and 1. Negative values indicated that the group with the lowest score answered the item randomly, and values close to 1 meant that the group answering the item showed a higher score compared to the knowledge about TB and, therefore, the item had greater discrimination^15^. 

In phase 3, the reliability of the instrument was assessed, which refers to the metric property that is related to the ability of the test of offering an estimate of the attribute magnitude with the least possible error, so that the result is as much closer to the reality of the phenomenon under study as possible, and can show the precision or consistency of the result obtained through the use of the instrument along several measurements^16^.

Instrument performance was analyzed through the responses of a sample of PTB patients, to define whether the variance of responses was related to the KAP construct of PTB patients about their illness^17^. A sample was taken from the universe of PTB patients under treatment in the TB Program of Bogotá from January 1 to December 31, 2016. Sample size was calculated for the population of 2017, with a 95% confidence interval and 5% error, assuming that the total number of the population in 2016 would evince a similar epidemiological behavior to that of 2017, as one could not know precisely how many patients would enroll to the TB Program during the study period. It is noteworthy that, in 2016, there were 1,327 cases of TB in Bogotá, 1,198 of which were new cases and 824 were PTB cases^3^. Considering there was no approximation of variance, the CFR MATA general sample size equation was used:

$$n\geq \frac{n}{(N-1)*K^{2}+1}$$

Where K is the margin of error (precision); N, the size of the study population; n is the sample size; so that the following results were found:

$$R=\frac{206}{824}=0,25$$

The sample size was 206 PTB patients newly admitted to the TB Control Program of Bogotá. Inclusion and exclusion criteria of PTB patients were the same already mentioned for the pilot comprehensibility test and for the sensitivity test for the group of PTB patients. 

Subsequently, the consistency of the responses in each of the components was analyzed through a factorial analysis of the 3 proposed dimensions; Cronbach’s alpha was used to analyze the internal consistency of the items and the reliability index, whose value allows us to estimate the percentage of possible error in the estimation of the attribute magnitude^18^.

A study of comprehensibility was developed with the same sample of PTB patients who participated in the reliability test and with experts’ judgement. Comprehensibility was evaluated from the reports of patients about the language, clarity, punctuation and writing, using a qualitative scale^19^. 

In phase 4, the evidence of content validity was obtained, so that an expert judgment was developed to conceptually support the measuring instrument. The judgement of experts assessed the criteria of sufficiency, clarity, relevance and coherence^20^; the answers of judges were consolidated and their valuation for the four criteria of interest was analyzed, through which the interquartile range of their responses was calculated. In cases where the difference is greater than 1.5, it indicates disagreement, so it was necessary to review the result differences for the four criteria of interest and the observations of the judges to develop adjustments to the items^21^.

The statistical analysis of the PTB patients’ responses to the items was performed to identify the average and standard deviation of the KAP levels of the patients^22^. Data of the research were collected between January and October 2017 and were later systematized and analyzed. 

Regarding ethical considerations, the research complied with the provisions of Resolution 8430 of the Ministry of Health and Social Protection of Colombia and was classified as a minimal risk since no physical or psychological modification was made to the participants, as only the instrument was applied to PTB patients and to the control group, after signing a written informed consent form^23^. The research was supported by the Ethics Committee of the School of Nursing of the National University of Colombia. 

## Results

Regarding the integrative review, 1,747 articles were found through the use of search equations with the aforementioned descriptors and Boolean operators. Subsequently, 34 articles whose full text was available were selected, mainly those that included the topic of KAP in PTB patients on their disease and those that were repeated were excluded. 

According to the SIGN level, 28 (82.3 %) articles were classified into level 3 (non-analytical studies such as clinical observations and case series), 1 (2.9 %) into level 2- (cohort or case-control studies with high risk of confusion, bias or chance, and a significant probability that the relationship is not causal), and 5 (14.7 %) into level 2+ (well-conducted cohort or case-control studies, with low risk of confusion, bias or chance, and a moderate probability that the relationship is causal)^14^. 

Likewise, of the 34 selected articles, 4 (12 %) were published in 2010, 3 (9 %) in 2011, 4 (12 %) in 2012, 2 (6 %) in 2013, 5 (15 %) in 2014, 10 (29 %) in 2015 and 6 (18 %) until August 31, 2016. Regarding the continent and/or region of publication, 11 (32 %) were developed in Asia, 10 (29 %) in Africa, 6 (18 %) in Latin America, 4 (12 %) in Europe, 2 (6 %) in North America and 1 (3 %) in Oceania. 

Then, a version of the instrument was devised, consisting of 3 dimensions and 43 items. Also, the instrument included a section of general information about the patient with TB, with 21 questions; a section of programmatic data on TB, with 10 questions; a section of programmatic data on TB and HIV, with 9 questions, and a section on TB information sources, with 4 questions. This version was subjected to a comprehensibility analysis with 30 PTB patients, and 7 items (2, 6, 9, 10, 12, 21 and 37) with a comprehensibility lower than 95% were found, which were adjusted according to the observations of patients with TBP, to facilitate their comprehensibility. The average comprehensibility of the knowledge dimension was 95.75 %; of the attitude dimension, 100 %; 96.6 % for the practice dimension, and 97.4 % on instrument comprehensibility. 

The sensitivity test was carried out with 30 PTB patients and 30 people forming a control group. In the group of patients with PTB, 12 (40 %) were adults (from 40 to 59 years of age), 11 (37 %) young adults (from 20 to 39 years old), and 7 (23 %) older adults (60 years or older); 20 (67 %) were male and 10 (33 %) female; 1 (3 %) did not have any level of education, 12 (40 %) had primary education, 14 (46 %) had high school education, 2 (7 %) had technician/technologist level, and 1 (3 %) had university education. Of the control group, 14 (47 %) were young adults, 13 (43.3 %) adults, 2 (6.6 %) older adults and 1 (3 %) was an 18-year-old teenager; 20 (67 %) were female and 10 (33 %) were male; 19 (63 %) had high school education, 9 (40 %) had primary education, 1 (3%)was a technician/technologist and 1 (3 %) had higher education. 

**Figure 2:**
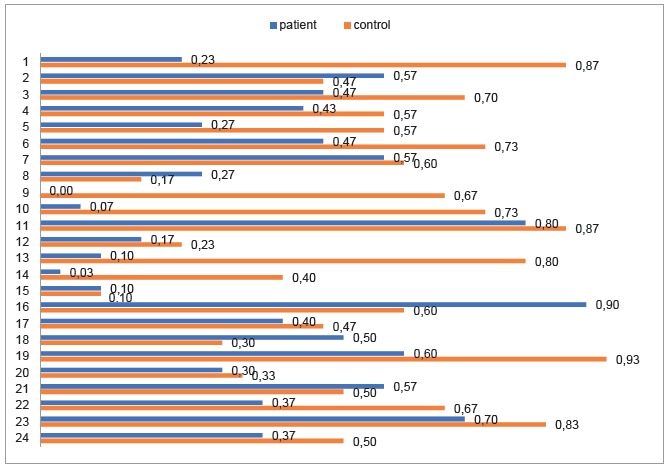
Difficulty of the questions about the knowledge dimension on pulmonary tuberculosis in the patient and control groups. Bogotá, Colombia, 2017

It was found that, of the 24 questions related to the dimension of knowledge on PTB, the average in the group of patients was 12.03 and, in the control group, 9.93. In the group of PTB patients, the average difficulty was 0.38, whereas in the control group it was 0.57. Details on the degree of difficulty of the 24 items of the dimension of knowledge on PTB are shown in Figure 2. 

**Figure 3:**
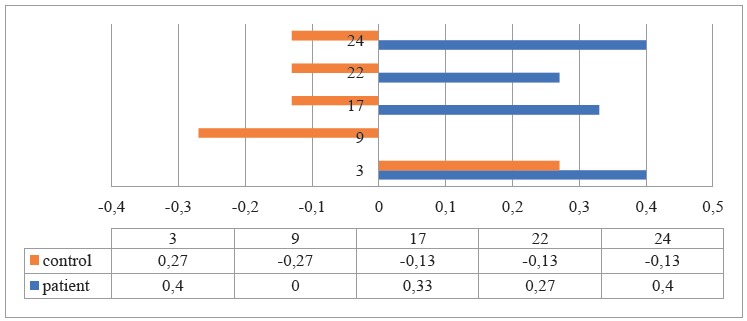
Discrimination of some items of the knowledge dimension on pulmonary tuberculosis in the patient and control groups. Bogotá, Colombia, 2017

Likewise, Figure 3 shows that in the group of patients the average of discrimination was 0.21 and, in the control group, 0.12, so it was found that some items were answered at random by the control group , and in the group of patients with TBP the discrimination was higher. 

234 patients with PTB participated in the reliability study, so that the sample size was exceeded by 13.5%: 149 (63.7%) men and 85 (36.3%) women; 17 (7.26 %) were between 18 and 19 years old, 81 (34.6 %) were young adults, 52 (22.2 %) adults and 84 (35.9 %) older adults; 167 (71.3 %) did not belong to any TB risk group, however, 30 (12.8 %) were homeless, 20 (8.6 %) were deprived of liberty, 10 (4.3 %) were health workers and 7 (3%) belonged to the indigenous population; 20 (8.5 %) had no formal education, 79 (33.7 %) completed only the primary level, 101 (43.1 %) went to high school, 19 (8.1 %) were technicians/technologists and 15 (6.5 %) had university education.

Factor analysis results showed 7 factors that explained 67.8 % of the total variance, which shows a structure consistent with conceptual approaches that defined the KAP dimensions. Knowledge on PTB is the main factor, which comprises 24 items; however, items 14 and 19 were placed in other factors. The second factor are the PTB practices, consisting of 10 items; items 31 and 37, however, were placed under other factors. Four items were grouped under the dimension of attitudes, but some of them have a low factor load. Table 1 shows the factor analysis results for the aforementioned dimensions. 

**Table 1:** Factor analysis of the dimensions of knowledge, attitudes and practices of the measurement instrument. Bogotá, Colombia, 2017

Item	Load	Factor	Cronbach’s Alpha
20	0.601	Knowledge on pulmonary tuberculosis*	0.65
5	0.574		
22	0.558		
9	0.546		
11	0.542		
7	0.498		
2	0.476		
12	0.466		
3	0.453		
8	0.453		
21	0.435		
18	0.431		
23	0.411		
17	0.395		
13	0.386		
6	0.382		
10	0.373		
1	0.335		
16	0.325		
4	0.324		
15	0.303		
33	0.793	Practices on pulmonary tuberculosis^†^	0.58
34	0.738		
29	0.597		
30	0.594		
32	0.441		
35	0.409		
38	0.351		
36	0.314		
28	0.504	Attitudes on pulmonary tuberculosis	0.32
27	0.337		
25	0.303		
26	0.164		

* Items 14 and 19 were not located under the knowledge factor.

† Items 31 and 37 were not located under the practices factor.

The same 234 PTB patients who took part in the reliability tests participated in it. One can see that the average comprehensibility of the instrument was 98.3 %; hence, the items were understandable for the population under study. In addition, adjustments were performed in some of the items according to the participants’ reports, as shown in Table 2. Items that were not described have obtained a 100% comprehensibility. 

**Table 2:** Comprehensibility percentages of the 234 patients with pulmonary tuberculosis on the instrument items. Bogotá, Colombia, 2017

Item	(%) I do not understand it	(%) I partially understand it	(%) I fully understand it
1	0	3	97
2	3	9	88
3	0	3	97
6	2	3	95
7	1	5	94
19	2	9	91
TOTAL	0.3	1.4	98.3

Three judges with expertise in the subject of TB and with professional and postgraduate academic training participated in the expert judgment: two members of the Colombian Anti-Tuberculosis League and one who worked in the TB Control Program of Bogotá. 

Regarding sufficiency, 37 (97.3 %) of the 38 items were consistent among the judges since they obtained a rating of 4, meaning the items that belong to this same dimension were sufficient to measure it. However, item 14 gave rise to discrepancies and its interquartile range was 1.5 because one of the judges considered such item did not contribute to the measuring of the dimension of knowledge on PTB, insofar as it is part of programmatic aspects facing treatment access, for which it was modified. 

As to clarity, the 38 (100 %) items were consistent among the judges, obtaining a score of 4, meaning, therefore, the syntax and semantics of the items was appropriate. 

Concerning relevance, 36 (94.7 %) of the 38 items were consistent among the judges since they obtained a rating of 4, which shows they were essential and should be included so to not affect the measurement of the dimension; nonetheless, in items 14 and 21 the interquartile range was 1.5, as one of the judges considered item 14 was not relevant, reason why it was modified, and other judge considered that item 21 should be included into other dimension, for which it was also modified. 

After the psychometric analysis for the sample of 234 PTB patients, the scale was defined through direct scoring, using as criterion the appropriate KAP on the patients’ PTB, and the levels were established considering the normative behavior of the sample. Four levels of KAP on PTB were established, namely: High (equal to or greater than 19), medium-high (14-18), medium low (9-13) and low (equal to or smaller than 8), which allowed classifying the KAP of PTB patients for the design and implementation of educational processes in Health. 

## Discussion

The KAP construct on the illness of PTB patients was developed from a integrative review in 6 databases, which allowed operationalizing the construct, and the items corresponded to the theoretical and conceptual development. To construct the items, we considered the collaborative TB/HIV actions promoted by the WHO. The average instrument comprehensibility was proved to be 97.4 % in the pilot test, and adjustments were made to the 6 items that showed a understandability below 95%. The instrument was shown to be sensitive for the population under study, so much that PTB patients attained an average score of 12.03, whereas the control group reached 9.93. 

However, in the comprehensibility study of content validity - conducted with 234 PTB patients, one can see an average item understandability of 98.3 %. In addition, some semantic adjustments were performed in the items that were not fully understandable. This shows the instrument is understandable for PTB patients in the city of Bogotá, and may require cultural and semantic adaptations to be replicated in other zones of Colombia and other countries, according to the characteristics of the population under study. 

In this methodological study, we identified that items 14, 19, 31 and 37 assessed specific aspects of TB that were not associated with any of the 3 KAP factors and, possibly, evidence new variables that should be reviewed in the future. Item 14 assessed the knowledge on the free access to anti-tuberculosis treatment, which many studies have identified as a programmatic knowledge that fosters the adherence to TB treatment and should be evaluated in the patients^24^ ^-^ ^25^; however, in the expert judgment, one of the judges considered that this item did not contribute to the measurement of the knowledge dimension and that it was not relevant, given that it referred to programmatic aspects of access to treatment. The item 19 sought to evaluate knowledge on adverse effects of the anti-tuberculosis drugs on patients, as it has been proved that some patients abandon treatment due to lack of knowledge on such adverse effects^26^.

Item 31 identified the practice of TB patients of hiding their diagnosis, as it had been observed they do so in their places of work and study for fear of being rejected, which can reduce adherence to treatment^27^. The item 37 evaluated whether TB patients currently used the surgical masks, aiming at crossing this information with a question of the section of programmatic data of the TB that described the phase and the number of doses of the treatment, given that the use of surgical mask by infectious TB patients is regarded as a strategy to avoid the transmission of the disease to other people^28^. 

The appropriate KAP of TB patients on their illness is a tool that can help reduce the transmission of this disease and wrong practices, decrease the self-medication and stigmatization, promote the adherence to anti-TB treatment, detect early contacts of TB patients who may have the disease and promote the demand for health services in new cases^29^. Therefore, an instrument with sensitive, reliable, and valid measures is a tool that can be applied to PTB patients in the city of Bogotá and may also be adapted for other scenarios with similar characteristics. 

Similarly, the Collective Health and Care Research Group of the School of Nursing of the National University of Colombia considers it important to develop instruments with reliable and valid measures on TB KAP of other population groups, such as health workers, community and family. 

## Conclusions

The KAP measuring instrument of PTB patients on their disease was constructed from an integrative review that supported the operationalization of the construct and includes empirical studies that support its sensitivity, reliability, and content validity. However, other psychometric studies are needed that analyze in greater depth items 14, 19, 31 and 37, which were not located in the dimensions related to the KAP. 

Finally, the Collective Health and Care Research Group of the School of Nursing of the National University of Colombia has developed studies on the topic of KAP on TB for more than 10 years and has identified the need to have instruments with sensitive, reliable, and valid measures. Therefore, the KAP instrument in PTB patients with constitutes an advance in the measurement of this phenomenon and will allow the development of other studies, of greater scope, in the city of Bogotá and in other scenarios, aimed at generating educational, political, economic and social interventions that allow transforming the KAP and, thus, contribute to the control of TB. 
